# Age-related changes in myelin and myelin water quantified with short-TR adiabatic inversion-recovery (STAIR) sequences

**DOI:** 10.1016/j.nicl.2025.103801

**Published:** 2025-05-09

**Authors:** James Lo, Jiaji Wang, Dylan Tran, Gabrielle Nemeh, Brandon Liu, Soo Hyun Shin, Jiyo S. Athertya, Dawn Schiehser, Yajun Ma, Jiang Du

**Affiliations:** a Department of Radiology, University of California, San Diego, CA, USA; b Department of Bioengineering, University of California, San Diego, CA, USA; c School of Medicine, University of California, San Diego, CA, USA; d Research Service, Veterans Affairs San Diego Healthcare System (VASDHS), San Diego, CA, USA; e Department of Psychiatry, University of California, San Diego, CA, USA; f Radiology Service, Veterans Affairs San Diego Healthcare System, San Diego, CA, USA

**Keywords:** Myelin content, Myelin water, Aging, Brain, Biomarker

## Abstract

Myelin proton density fraction (MPDF) and myelin water fraction (MWF) measured with short-TR adiabatic inversion-recovery (STAIR) sequences are potential biomarkers of aging and neurodegeneration, but the effects of aging on MPDF/MWF remain unknown. This study aims to assess relationships between age and MPDF/MWF using the 3D STAIR ultrashort echo time (STAIR-UTE) and STAIR short echo time (STAIR-STE) sequences, respectively. 42 volunteers (29 young (<55y), 13 old (>55y)), were recruited for MPDF and MWF mapping for white matter (WM) and gray matter (GM) on a 3 T scanner. Excellent inter-reader reliability was demonstrated for MPDF and MWF measurements with ICC values of 0.97 and 0.98, respectively, between two readers. In the young group, WM MPDF and MWF range from 8-13 % and 6–13 %, respectively, while GM MPDF and MWF range from 5-7 % and 3–5 %, respectively. In the old group, WM MPDF and MWF range from 6-12 % and 5–13 %, and GM MPDF and MWF range from 3-6 % and 2–5 %, respectively. The young group’s MPDF/MWF values were significantly higher than those of the old group. Altogether, the 42 volunteers display linear and quadratic associations of MPDF/MWF with age. MPDF demonstrated significant correlations with MWF in the majority of brain regions. This study demonstrates the capability of two myelin imaging biomarkers, STAIR-UTE measured MPDF and STAIR-STE measured MWF to map brain myelin and MW. These biomarkers hold the potential to differentiate normal aging from neuroinflammatory/neurodegenerative diseases.

## Highlights

• STAIR-UTE maps myelin and STAIR-STE maps myelin water in young and old volunteers.
• High ICC values demonstrate excellent inter-reader reliability of the protocol.
• MPF and MWF maps showed significant differences between young and old volunteers.
• Most WM/GM regions show significant quadratic and linear relationships with age.
• WM and GM regions demonstrate significant correlations between MPF and MWF.

## 1 Introduction

The annual growth in the elderly population worldwide has substantially increased the global burden of age-related diseases (Behr et al., 2023, Partridge et al., 2018). Dementia and related neurodegenerative conditions have become increasingly prevalent diseases among the older population (Wyss-Coray, 2016). Previous studies suggest the deterioration of myelin integrity may play a crucial role in both the onset and progression of cognitive impairment and dementia (Bouhrara et al., 2018, Mercier et al., 2024, Alonso-Ortiz et al., 2015).

The myelin sheath, an extension of oligodendrocyte membranes comprised of tightly arranged lipids, proteins, and water, envelops segments of axons to enhance the speed and efficiency of neural signal transmission (Barkovich, 2000). Myelin development begins in early childhood and increases with age throughout the brain, but especially in particular regions, such as the parietal, temporal, cerebellar, and occipital cortices (Lee et al., 2015). The myelination process reaches its peak approximately between the third and fifth decade of life, and then the myelin content precipitously decreases in old age (Benes et al., 1994, Wu et al., 2016, Grydeland et al., 2013). This trajectory follows the well-established inverted-U, quadratic relationship that is found between normal aging and myelin content, and is consistent with the principle of retrogenesis (Grydeland et al., 2013, Yeatman et al., 2014, Qian et al., 2020, Bartzokis et al., 2012, Arshad et al., 2016). Multiple hypotheses are consistent with retrogenesis, namely the “gain-predicts-loss” hypothesis, which proposes that the rate of change in brain development is mirrored in decline: the more gained before the peak, the more lost after (Yeatman et al., 2014). Alternatively, the “last-in-first-out” hypothesis states that the latest maturing tissue and regions of the brain are especially vulnerable to deterioration in old age, leading to aging-associated degeneration to occur in the reverse order of development-associated maturation (Yeatman et al., 2014, Heath et al., 2018, Brickman et al., 2012). Distinguishing the behavior of myelin in normal age-related changes from that of disease-related pathologic changes provides useful information about the mechanisms of cognitive impairment and illuminates the principle of retrogenesis further. Consequently, an accurate in vivo measurement of the myelin sheath has become essential for assessing cognitive function in the elderly (Dimovasili et al., 2023).

MRI provides non-invasive methods for measuring the signals of non-aqueous myelin and myelin water (MW). Diffusion-weighted imaging (DWI) and diffusion tensor imaging (DTI) utilize the diffusion patterns of water within the axons to explore myelin integrity and content (Yeatman et al., 2014, Inano et al., 2011, Westlye et al., 2010). On the other hand, techniques like magnetization transfer (MT) imaging and T2 relaxation time methods (e.g., multi-echo spin-echo (SE) T_2_ relaxation acquisition and multi-component Driven-Equilibrium Single-Pulse Observation of T1 and T2 (mcDESPOT)) probe the trends of myelin change with age by measuring MW (Arshad et al., 2016, Wu et al., 2016, Bouhrara et al., 2020). Such techniques have demonstrated sensitivity to the demyelinated lesions of multiple sclerosis (MS) (MacKay and Laule, 2016). They have also been used to establish the inverted-U, quadratic relationship between normal aging and myelin content (Qian et al., 2020).

However, each technique has its limitations. Diffusion imaging assesses myelin integrity by measuring various diffusion parameters (e.g., fractional anisotropy (FA), axial diffusivity (AD), radial diffusivity (RD)), but subtle myelin thinning without major structural disruption may remain undetected. Thus, DWI/DTI effectively identifies acute myelin damage but may underestimate loss when myelin is thinner without rupture, even though myelin content is decreased (Fieremans et al., 2011, Song et al., 2005). Furthermore, diffusion measurements are also influenced by axon density, diameter, membrane permeability, fiber direction consistency, and non-myelin factors like gliosis and increased intercellular spacing (Groeschel et al., 2016, Zhang et al., 2020). MT imaging, which infers macromolecular proton content, is affected by various factors beyond myelin, such as the presence of other macromolecules and paramagnetic compounds like iron (Zhang et al., 2020, Todorich et al., 2009, Prevost et al., 2021). The traditional multi-echo SE T2 relaxation acquisition method for MW imaging, although correlating well with histology, suffers from long acquisition times, limited coverage, and inaccurate MW fraction (MWF) estimates due to edema, iron accumulation, MT effect, and other factors (Alonso-Ortiz et al., 2015, Barta et al., 2015, Bjarnason et al., 2005). While more time efficient, the mcDESPOT technique has similar limitations (Lankford and Does, 2013, Zhang et al., 2015). Furthermore, the techniques above target indirect biomarkers of myelin (or MW), rather than direct imaging of myelin (or MW) itself.

A major challenge for direct imaging of myelin is the extremely short T_2_s of myelin protons (<1 ms) (Horch et al., 2011, Ma et al., 2022), which is significantly shorter than the echo times (TE) of conventional sequences (typically ranging from several to tens of milliseconds). As a result, the myelin signal has already decayed to near zero by the time conventional MR data acquisition is initiated, making it difficult to directly image myelin and accurately assess age-related changes in myelin content. Compounding this issue is the signal contamination from intra/extracellular water (IEW) components, which have significantly higher proton density (PD) and longer T_1_ and T_2_ than non-aqueous myelin or MW, leading to an overestimation of myelin content (Ma et al., 2022, Ma et al., 2020, Ma et al., 2020).

Recently, the short-TR adiabatic inversion recovery (STAIR) contrast technique has been developed to measure myelin and MW content accurately (Ma et al., 2022, Ma et al., 2020, Ma et al., 2020, Ma et al., 2022). This sequence is characterized by achieving high-efficiency suppression of signals from IEW components with longer T_1_/T_2_ than those of myelin or MW by combining an optimized inversion time (TI) and short TR (Ma et al., 2020). Additionally, this method employs 3D Cones sampling for k-space filling, which reduces partial volume artifacts and enhances the spatial resolution of the imaging compared to traditional 2D sampling, further improving the visualization of myelin structures (Ma et al., 2020). The combination of the STAIR and ultrashort echo time (STAIR-UTE) sequence enables direct myelin proton density fraction (MPDF) mapping (Ma et al., 2020), while the combination of the STAIR and short echo time (STAIR-STE) sequence allows direct MW fraction (MWF) mapping (Ma et al., 2022). These biomarkers have shown potential clinical utility as significantly lower MPDFs and MWFs have been found in patients with MS both in lesions and normal-appearing white matter (NAWM) compared with normal white matter (NWM) in healthy controls (Ma et al., 2020, Ma et al., 2022, Lo et al., 2024).

In this cross-sectional study, we aim to investigate age-related changes in myelin and MW content across a broad age range (20-87y) of healthy adults using a 3 T clinical scanner. Relative MPDF and MWF were measured in various white and gray matter regions. Both myelin biomarkers were compared between younger (<55 years) and older (>55 years) adults to evaluate the trajectory of myelin changes with age.

## 2 Methods and materials

### 2.1 Pulse sequences

The STAIR-UTE sequence employs an adiabatic full passage (AFP) pulse to invert the longitudinal magnetization of all brain water components, including the MW and IEW. The magnetization of non-aqueous myelin, with its rapid transverse magnetization decay (∼0.2 ms) (Ma et al., 2020, Sheth et al., 2016), is saturated rather than inverted. The UTE data acquisition with an ultrashort TE of 0.032 ms begins at an optimal TI when the inverted water components recover to their nulling point, suppressing their off-target signal contributions and enabling specific myelin detection.

The STAIR-STE sequence uses a similar method of long T_2_ component signal suppression as STAIR-UTE, but is specific for MW and its relatively short T_2_ (∼10 ms) (Ma et al., 2020, Ma et al., 2022, Kim et al., 2015, Oh et al., 2013, Hwang et al., 2010, Mackay et al., 1994, Chan et al., 2023). Following the AFP pulse, the longitudinal magnetization of MW recovers faster than that of IEW due to its shorter T_1_. The STE sequence employs a longer TE (i.e., TE = 0.8 ms) to allow the shorter T2* myelin signal to decay to near zero, while preserving that of MW (Ma et al., 2022). This enables robust suppression of both long T_2_ IEW signal and ultrashort T_2_ myelin signal and thus selective imaging of MW.

As such, specific TEs of 0.032 and 2 ms are used in the STAIR-UTE/PD-weighted UTE (PD-UTE) and STAIR-STE/PD-weighted STE (PD-STE) sequences, respectively. These TEs in conjunction with sufficiently short TRs (110 and 250 ms, respectively) enable long T_2_ water signal suppression and selective imaging of non-aqueous myelin in STAIR-UTE and MW in STAIR-STE. The detailed sequence parameters in this study are listed in Table 1.

**Table 1:** Sequence parameters for the imaging protocol, including STAIR-UTE and PD-UTE to map MPDF, and STAIR-STE and PD-STE to map MWF.

**STAIR-UTE-Radial**	**PD-UTE-Radial**
FOV = 23 × 23 × 42 cm^3^, voxel size = 2.13 × 2.13 × 3.5 mm^3^, TR/TI = 110/53 ms, TE = 0.032 ms, N_sp_ = 5, τ = 5.4 ms, FA = 15°, bandwidth = 125 kHz, scan time = 9 min 24 s	FOV = 23 × 23 × 42 cm^3^, voxel size = 2.13 × 2.13 × 3.5 mm^3^, TR/TE = 7/0.032 ms, FA = 1°, bandwidth = 125 kHz, scan time = 1 min 11 s
**STAIR-STE-Cones**	**PD-STE-Cones**
FOV = 23 × 23 × 14.4 cm^3^, voxel size = 1.44 × 1.44 × 4 mm^3^, TR/TI = 250/114 ms, TE = 2 ms, N_sp_ = 5, τ = 5.4 ms, FA = 40°, bandwidth = 83.3 kHz, scan time = 9 min 57 s	FOV = 23 × 23 × 14.4 cm^3^, voxel size = 1.44 × 1.44 × 4 mm^3^, TR/TE = 7/2 ms, FA = 1°, bandwidth = 83.3 kHz, scan time = 1 min 34 s

### 2.2 MR data acquisition

Twenty-nine young (<55y) normal volunteers (aged 29 ± 9 years old, 21 female) and thirteen old (>55y) normal volunteers (aged 70 ± 8 years old, 9 female) were recruited and scanned. Written informed consent was obtained from each participant as approved by the institutional review board (IRB) of the University of California, San Diego, with registration number 800639. All participants underwent scanning with a 3 T clinical scanner (MR750; GE Healthcare, Milwaukee, Wis), utilizing a 12-channel receive-only head coil for signal reception.

Clinical MRI sequences are included in the imaging protocol, namely T_1_ fast spoiled gradient echo (FSPGR) BRAVO images and T_2_ fluid-attenuated inversion recovery sequences (FLAIR), were employed to exclude subjects with abnormal pathology (e.g., tumors, post-ischemic defects, etc.). However, normal aging associated leukoaraiosis, with the criteria of Fazekas score ≤ 1, did not exclude any subjects from the study (Fazekas et al., 1987).

For investigation, the imaging protocol includes STAIR-UTE, proton-density weighted UTE (PD-UTE), STAIR-STE, and PD STE (PD-STE) sequences. MPDF and MWF were calculated as the ratio of STAIR-UTE signal over PD-UTE signal and the ratio of STAIR-STE signal over PD-STE signal, respectively.

### 2.3 Data analysis

A histogram of the age distribution was generated to better describe the demographics. MPDF and MWF maps were generated using MATLAB software (Mathworks Inc., Natick, Mass). Regions of interest (ROIs) were delineated by two experienced, blinded radiologists on eight white matter (WM) regions: the left and right centrum semiovales, periventricular regions, subcortical WM, and splenium and genu of the corpus callosum, and two grey matter (GM) regions: the putamen and thalamus.

### 2.4 Statistical analysis

The intraclass correlation coefficient (ICC) was calculated for inter-reader agreement between all MPDF and MWF measurements using SPSS software (IBM, Armonk, NY, USA). Statistical differences between the young and old groups were assessed by first averaging each subject’s 8 WM and 2 GM ROIs into composite WM and GM values. Group comparisons were then performed using Mann-Whitney *U* test in GraphPad Prism (GraphPad Software, Boston, MA, USA). Additionally, both linear and nonlinear quadratic regression were performed to evaluate the relationship between MPDF/MWF in each region and age. P-values were derived using an extra sum-of-squares F test comparing the quadratic model to a null (horizontal line) model, the linear model against the null model, and the quadratic model to the linear model. Pearson’s correlations were also calculated to evaluate the relationships between MPDF and MWF. R values were interpreted as: 0–0.29, negligible; 0.3–0.49, low; 0.5–0.69, moderate; 0.7–0.89, high; 0.9–1.0, very high (Hinkle et al., 2003).

## 3 Results

### 3.1 MPDF and MWF maps

**Fig. 1:**
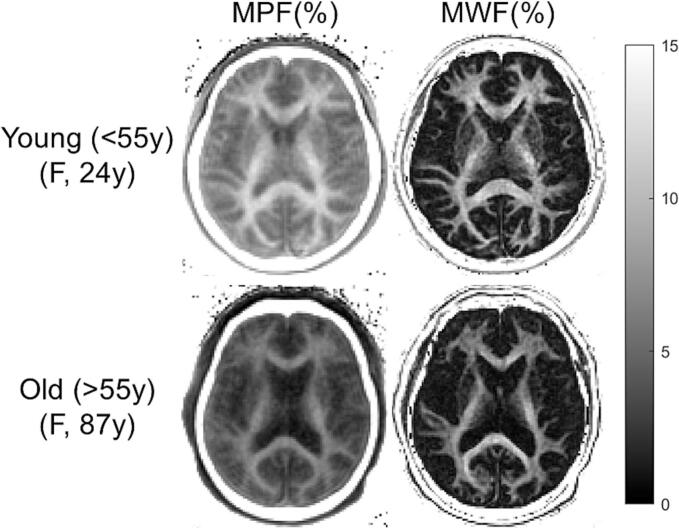
Representative MPDF (left column) and MWF (right column) maps comparing a 24-year-old volunteer (young group(<55y)) (top row) with an 87-year-old volunteer (old group (>55y)) (bottom row). WM regions display higher MPDF and MWF than GM regions, consistent with WM being more myelin-rich than GM. The young volunteer displays noticeably higher MPDF and MWF than the old volunteer.

Excellent repeatability of the protocol was demonstrated with high ICC values (0.97 and 0.98 for MPDF and MWF ROI measurements, respectively) between two readers. Fig. 1 shows the representative MPDF and MWF maps comparing a 24-year-old volunteer with an 87-year-old volunteer. WM regions display higher MPDF and MWF than GM regions, consistent with higher myelin content in white matter regions. The young volunteer displays noticeably higher MPDF and MWF throughout the whole brain than the old volunteer.

### 3.2 Comparison between young and old normal volunteers

**Fig. 2:**
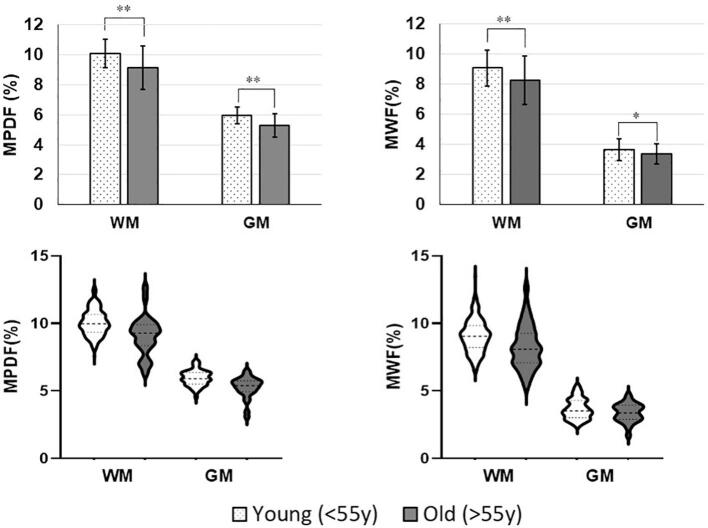
Bar plots (top row) comparing the averaged WM and GM (left column) MPDF and (right column) MWF of the young (<55y, n = 29) group and the old (>55y, n = 13) group. Mann-Whitney *U* test analysis was performed on WM and GM ROI values averaged by subject to investigate statistical difference between the young and old groups (“*” indicates *p* < 0.05, “**” indicates *p* < 0.01). MPDF and MWF measurements in the WM and GM of the young group are significantly higher than those of the old group. Corresponding violin plots (bottom row) of ROI values for the MPDF (left column) and MWF (right column) to visualize group distributions. WM and GM from the young group are depicted in dotted white. WM and GM from the old group are depicted in grey.

Supplemental Fig. 1 shows the histogram of the age distribution, with a concentration of individuals below 30 years old. Fig. 2 shows the bar plots comparing the averaged WM and GM MPDF(%) and MWF(%) of the young (<55y, n = 29) group and old (>55y, n = 13) group as well as their corresponding violin plots. In the young group, WM MPDF and MWF range from 8–13 % and 6–13 %, respectively, while GM MPDF and MWF range from 5–7 % and 2–5 %, respectively. In the old group, WM MPDF and MWF range from 6–13 % and 5–13 %, and GM MPDF and MWF range from 3–6 % and 2–5 %, respectively. MPDF and MWF measurements in the WM and GM of the young group are significantly higher than those of the old group.

### 3.3 Quadratic behavior of MPDF and MWF with age

**Fig. 3:**
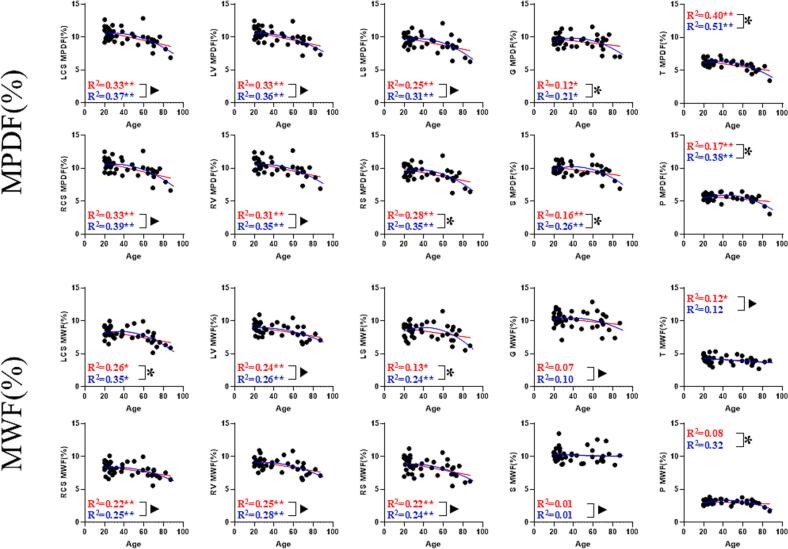
Scatterplots for all MPDF (top two rows) and MWF (bottom two rows) ROI measurements for each region (LCS/RCS = left/right centrum semiovale; LV/RV = left/right periventricular region; LS/RS = left/right subcortical WM; G = genu; S = splenium; P = putamen; T = thalamus) with age across all 42 young and old group volunteers. Each plot displays fitted regression lines: red for the linear model and blue for the quadratic model, with their corresponding coefficients of determination R^2^ and p-values when compared to the null model (p < 0.05, denoted by a “*”, and p < 0.01, denoted by a “**”). Black brackets indicate comparisons between the quadratic and linear models. (p > 0.05, denoted by a “▲”, p < 0.05, denoted by a “*”).

Fig. 3 shows the linear and quadratic curves for MPDF and MWF ROI measurements of each region across all 42 participants from young (<55y) and old (>55y) volunteer groups. Curves generally follow quadratic regression and linear decrease with increasing age. Significance was determined by an extra-sum-of-squares F test comparing: the quadratic model to a null model (horizontal line), the linear model to a null model, and the quadratic model to the linear model. All regions demonstrated significantly better fit with both the quadratic and linear models compared to the null model, except those of the G/S/T/P regions of the MWF. Notably, only the MPDF G/RS/S/T/P and MWF LS/RCS/P demonstrated significantly improved fitting with the quadratic models over linear models.

### 3.4 Correlation between MPDF and MWF

**Fig. 4:**
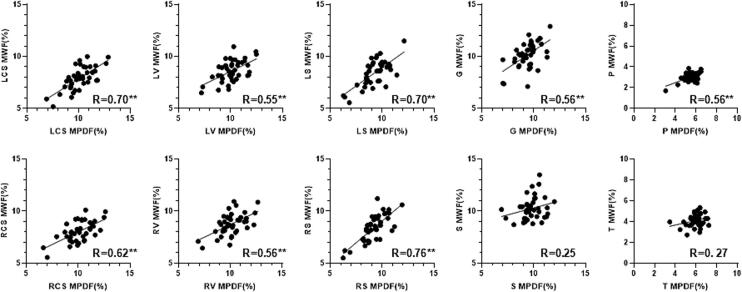
Linear regression lines and scatterplots for MPDF and MWF measurements for each region (LCS/RCS = left/right centrum semiovale; LV/RV = left/right periventricular region; LS/RS = left/right subcortical WM; G = genu; S = splenium; P = putamen; T = thalamus) across all 37 young and old group volunteers. All Pearson’s correlation R values are displayed in the bottom right of each graph and exhibit positive correlations between MPDF and MWF. All correlations except that of S MWF vs. MPDF are significant (p < 0.05, denoted by a “*” and p < 0.01, denoted by a “**”).

Fig. 4 shows the linear regression lines and scatterplots for the correlation between MPDF and MWF measurements for each region across all 42 volunteers. Pearson’s correlation R values are moderate to high in the WM, except for S (splenium), and moderate in GM except for T (thalamus). All correlations except those of S (splenium) and T (thalamus) MWF vs. MPDF are significant.

## 4 Discussion

In this cross-sectional study, we investigated the relationships between STAIR-UTE measured MPDF and STAIR-STE measured MWF with age in several WM and GM regions in a healthy adult population spanning a wide range of ages. Strong inter-reader correlations were found between all MPDF and MWF measurements for all subjects. The young group displayed significantly higher MPDF and MWF in WM and GM regions than the old group. Our results indicate that in the majority of brain regions analyzed, both the MPDF and MWF have weak but significant quadratic and linear relationships with age. Furthermore, MPDF and MWF show moderate to high correlations in WM, except the splenium region, and low to moderate correlations in GM, across all 42 volunteers.

As direct myelin MRI quantification is still in its infancy, previous attempts to associate myelin with age have been based on indirect indicators of myelin, such as changes in MT ratio (MTR), or T_1_ relaxation time, or DTI (Qian et al., 2020, Bouhrara et al., 2020, Callaghan et al., 2014, Bartzokis, 2011, Westlye et al., 2010, Billiet et al., 2015). Limited success has been achieved with inversion recovery ultrashort echo time (IR-UTE) sequences (Sheth et al., 2016, Waldman et al., 2003, Wilhelm et al., 2012, Du et al., 2014), in which UTE data acquisition with TEs (<0.1 ms) is used to detect myelin signals. Meanwhile, signals from IEW components are suppressed by adiabatic inversion preparation and echo subtraction. The 3D zero TE (ZTE)-based hybrid filling (HYFI) technique also achieves myelin imaging by combining single-point imaging with multiple radial readouts at lowered gradient strength to fill the gap in the central k-space created by RF dead time (Weiger et al., 2020). Inhomogeneous MT (ihMT) has also demonstrated myelin specificity by selectively targeting the long dipolar relaxation time (T1D) unique to the ordered structure of multiple lipid bilayer myelin to distinguish it from WM (Girard et al., 2015, Alsop et al., 2023). The ihMT signal is derived from the difference between conventional MT signals (single off-resonance saturation) and signals obtained using dual symmetric off-resonance saturation at equivalent RF power (Munsch et al., 2021, Duhamel et al., 2019). Quantitative MT (qMT) imaging uses multicompartmental models obtained with varied saturation power and offset to describe MT effects, specifically isolating the macromolecular proton fraction as an indicator of myelin content (Lee et al., 2021, York et al., 2022). Similarly, advances in MW imaging have been developed, like the T_2_/T_2_* relaxation approach that models heterogeneous brain tissue to separate two or more water compartments based on T_2_ and T_2_* differences with intensive modeling and postprocessing (Hwang et al., 2010, Mackay et al., 1994, Chan et al., 2023). Alternatively, the visualization of short transverse relaxation time components (ViSTa) technique suppresses long T_1_ signals with a double IR (DIR) to selectively image the short T_1_ MW without such postprocessing (Kim et al., 2015, Oh et al., 2013). These techniques have proven sensitive to MS-associated demyelination (Kim et al., 2015, Oh et al., 2013, MacKay and Laule, 2016). While STAIR-UTE measured MPDF cannot be directly compared to such metrics, all MPDF measurements of WM and GM regions demonstrate significant, quadratic and linear associations with age.

Our STAIR-STE measured mean MWF values in WM regions were overall in good agreement with those reported in previous studies (Oh et al., 2013, Qian et al., 2020, MacKay and Laule, 2016, Faizy et al., 2020). However, we note that our MWF value ranges (5–13 %) are somewhat lower than those obtained by mcDESPOT (11–27 %) or multiple SE techniques (8–17 %) (Zhang et al., 2015, Qian et al., 2020). This discrepancy can likely be attributed to factors including, but not limited to, MT effects between macromolecules and free water protons, exchange between water pools, T1 effect, J-coupling, off-resonance, spin locking, water diffusion within different compartments, and internal gradients, many of which may result in the overestimation of MWF (Qian et al., 2020). Nevertheless, our results indicate a similar significant quadratic association between MWF and age, not only in the WM regions but also in GM putamen (Qian et al., 2020, Bouhrara et al., 2020). Although statistical significance was not achieved in all brain structures, our STAIR-STE measured MWF with selective MW detection results corroborate the increase in myelin content from young to middle age and its subsequent decrease in old age established in the literature.

The accurate determination of myelin/MW content in GM is inherently more challenging than in WM. WM is composed of long myelinated axons that appear more spaced out and have a coarser texture than GM, which consists of densely packed neuronal cell bodies and dendrites. WM has significantly higher myelin content than GM (Bouhrara et al., 2018, Qian et al., 2020, Bonny et al., 2020), producing much higher signal-to-noise ratio (SNR) in STAIR-UTE and STAIR-STE images, thus more accurate MPDF/MWF measurements. The finer structure in GM also leads to increased partial volume artifacts, somewhat compromising MWF and especially MPDF measurements. Furthermore, not all cell types in the brain such as oligodendrocytes, microglia, astrocytes, etc., may change in a manner that adheres to the principle of retrogenesis as myelin does (Yeatman et al., 2014). The interplay between this variety of different brain cells and their development/degeneration that occurs with age is still under investigation, and cannot be fully described by solely monitoring myelin (Pang et al., 2013, De Waard and Bugiani, 2020). Accordingly, while our data is consistent with the “gain-predicts-loss” hypothesis, as seen in our U-curves between MPDF/MWF and age, it does not suggest region-specific degeneration differences in timing, which does not support the “last-in-first-out” hypothesis. Our findings present an important nuance: the majority of brain regions do not demonstrate significantly improved fitting of quadratic versus linear models, suggesting more uniform trajectories across structures as opposed to staggered degeneration timelines. In the case of strict ‘last-in-first-out” sequenced degeneration, divergent model fits would be expected (e.g., quadratic in more vulnerable regions versus linear in resilient ones).One potential factor that may cause this is the 10 brain structures analyzed do not show such pronounced differences in regional MPDF/MWF changes and changes may be more obvious in cortical GM rather than subcortical GM and WM, which is difficult to achieve with STAIR-UTE and STAIR-STE. Furthermore, our limited sample size, while sufficient to observe global quadratic and linear trends, lacks the statistical power and well-balanced age groups required to illuminate more subtle, region-specific temporal trends.

MWF is a well-established biomarker for assessing myelin content, traditionally measured via techniques such as mcDESPOT and multi-echo SE. Our employed STAIR-STE technique for quantifying MWF shows tentative promise with comparable results to these methods (Qian et al., 2020, Bouhrara et al., 2020), without requiring the aforementioned complicated modeling or intensive post-processing, despite similar acquisition times (∼10 min for STAIR-STE, ∼10–21 min for mcDESPOT) (Bouhrara et al., 2020, Dvorak et al., 2021, Bouhrara and Spencer, 2017). While comprehensive validation through immunohistological correlation and repeatability/reproducibility studies remains necessary to fully establish the robustness of STAIR-STE, our current findings support its reliability.

Our results show that all WM regions, except for the splenium and thalamus, displayed significant moderate to high correlations between MPDF and MWF, while GM regions displayed significant moderate correlations. These correlations suggest that STAIR-STE measured MWF, and by extension, STAIR-UTE measured MPDF, are reliable as measures of myelin and MW content. Together, these findings support the use of MWF and MPDF as complementary biomarkers in clinical and research settings, with STAIR-based techniques providing a reliable means for quantification.

There were several limitations in this study. First, the accuracy of our STAIR-UTE measured MPDF and STAIR-STE measured MWF was not verified using an external standard. Thus, the information presented in this study is largely semi-quantitative and further validation studies are warranted for these techniques. Second, the MPDF and MWF ranges in WM regions across both young and old groups are quite similar (6–13 % and 5–13 %, respectively), while these in GM regions are more distinct (3–7 % and 2–5 %, respectively). This can be attributed to image blurring caused by semisolid myelin-associated rapid T_2_ signal decay and limited gradient performance of a clinical MR system. Consequently, the ultrashort T_2_ myelin signal necessitates the STAIR-UTE protocol to employ a lower spatial resolution than that of the MW-specific STAIR-STE to maintain sufficient SNR for reliable quantification. This can artificially inflate the values of GM ROIs in the calculation of the MPDF maps, an effect absent in the MWF maps (Ma et al., 2021). This blurring is also exacerbated by myelin specific STAIR-UTE protocol Future studies could utilize a higher performance gradient system to mitigate this ultrashort T_2_ blurring effect and improve the quantification accuracy (Froidevaux et al., 2020). Third, while both the young and old cohorts spanned a wide age range, it did not include adolescent participants (<18 years old) in accordance with the inclusion and exclusion criteria of the IRB protocol of the University of California, San Diego, registration number 800639. The inclusion of such younger participants may affect the quadratic behavior of the MPDF/MWF by region and thus limit the generalizability of our findings (Qian et al., 2020, Fjell et al., 2010). Fourth, we note that participants between the third and fourth decade of life were not well-represented in our young cohort. As this is the age range where myelin and MW are expected to reach their peaks in healthy adults, this again may affect the quadratic behavior of the MPDF/MWF with age (Qian et al., 2020, Fjell et al., 2010). Fifth, our study does not stratify subjects by sex, race, education level, socioeconomic status, or other factors that may affect brain development and thus myelination behaviour (Klingberg et al., 2022, Ramzanpour et al., 2023). More detailed grouping may reveal more distinct sub-population specific myelination trends, rather than the generalized patterns observed in our current analysis. Sixth, our study only analyzed a cross-sectional dataset, so the true trajectories of the MPDF and MWF and regional vulnerabilities with age warrant further investigation through longitudinal studies with larger, age-group balanced cohorts. Seventh, MPDF and MWF are highly correlated, while their signal sources are distinctly different. This high correlation is likely due to the strong connection between myelin and MW. Further research is needed regarding the advantages and disadvantages of MPDF over MWF. Eighth, while this study demonstrates the potential of STAIR-based MPDF and MWF as biomarkers for age-related changes, the modest sample size, though sufficient to detect significant associations, precludes definitive conclusions about clinical significance. As such, these findings should be interpreted as preliminary evidence supporting MPDF and MWF, warranting future studies with larger, more diverse populations across varying degrees of neurodegeneration.

## 5 Conclusion

The STAIR-UTE measured MPDF and STAIR-STE measured MWF show significant differences in both WM and GM regions of the brain between young and old groups. Both MPDF and MWF show largely significant, quadratic and linear relationships, as well as negative correlations, with age in most brain regions, which holds potential in differentiating normal aging from neurodegenerative disease-associated abnormal aging.

## Funding information

The authors acknowledge grant support from the National Institutes of Health (RF1AG075717, R01AR079484, and F32AG082458), VA Clinical Science Research and Development Services Merit Award (I01CX002211), and GE Healthcare.

## CRediT authorship contribution statement

**James Lo:** Writing – review & editing, Writing – original draft, Visualization, Validation, Supervision, Methodology, Investigation, Formal analysis, Data curation, Conceptualization. **Jiaji Wang:** Writing – review & editing, Writing – original draft, Visualization, Validation, Formal analysis. **Dylan Tran:** Visualization, Validation, Formal analysis. **Gabrielle Nemeh:** Validation, Formal analysis. **Brandon Liu:** Validation, Formal analysis. **Soo Hyun Shin:** Writing – review & editing. **Jiyo S. Athertya:** Writing – review & editing, Funding acquisition, Formal analysis. **Dawn Schiehser:** Writing – review & editing. **Yajun Ma:** Writing – review & editing, Validation, Funding acquisition. **Jiang Du:** Writing – review & editing, Writing – original draft, Visualization, Validation, Supervision, Resources, Project administration, Methodology, Funding acquisition, Conceptualization.

## Declaration of competing interest

The authors declare that they have no known competing financial interests or personal relationships that could have appeared to influence the work reported in this paper.

## Data Availability

Data will be made available on request.

## Appendix A Supplementary data

Supplementary Figure 1 Histogram of age distribution of all subjects (n=42), with a concentration of individuals below 30 years old.
