# Supplementary figures and images for: Age-related changes in myelin and myelin water quantified with short-TR adiabatic inversion-recovery (STAIR) sequences
Source: Neuroimage Clin. 2025 May 9;46:103801. doi: 10.1016/j.nicl.2025.103801 (PMC12140967; doi:10.1016/j.nicl.2025.103801)

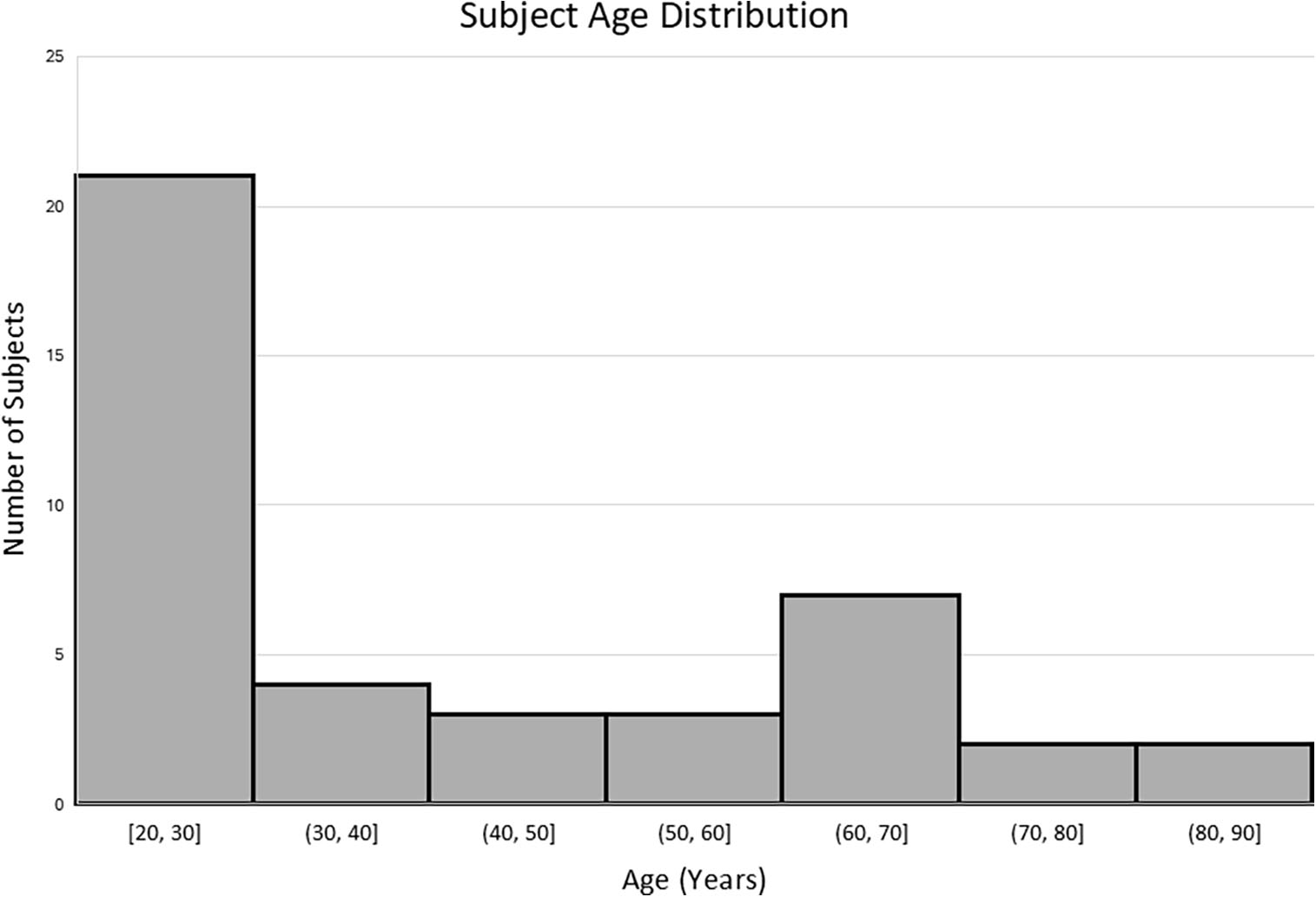

Supplement: Supplementary Figure 1 — Histogram of age distribution of all subjects (n=42), with a concentration of individuals below 30 years old. [file mmc1.jpg]
